# The key to our future lies in the past: How to integrate historical–geomorphological knowledge for future coastal decision-making?

**DOI:** 10.1017/cft.2026.10029

**Published:** 2026-04-23

**Authors:** Diana Giebels, Alexander Bartholomä, Jelle Bulens, Friederike Bungenstock, Frances E. Dunn, Holger Freund, Mikkel Fruergaard, Wienke Harms, Martina Karle, Kathrine Maxwell, Jaap Nienhuis, Minne Oostra, Monzer Sarker, Frans Sijtsma, Annika Traue, Achim Wehrmann

**Affiliations:** 1 Institute of Chemistry and Biology of the Marine Environment, University of Oldenburg, Wilhelmshaven, Germany; 2 Senckenberg am Meer, Marine Research Department, Wilhelmshaven, Germany; 3 Department of Physical Geography, Utrecht University, Utrecht, Netherlands; 4 Lower Saxony Institute of Historical Coastal Research, Wilhelmshaven, Germany; 5 Department of Geosciences and Natural Resource Management, Geography, Land, Environment and Society, University of Copenhagen, Copenhagen, Denmark; 6 Faculty of Spatial Sciences, University of Groningen, Groningen, Netherlands

**Keywords:** climate change adaptation, coastal morphodynamics, coastal sediment dynamics, coastal zone management, ecosystem-based management, coastal policy

## Abstract

Coastal areas that are characterized by coastal squeeze are highly vulnerable to the contemporary threats of climate change, sea level rise and biodiversity loss. Innovative coastal adaptation policies are necessary to accommodate these threats and have become salient issues on the agendas of mandated decision-makers on a global scale. This article provides a perspective on how historical–geomorphological knowledge can be integrated as an evidence base for coastal adaptation in such vulnerable zones when re-thinking and re-designing coastal adaptation policies. Our historical–geomorphological perspective proposes that we explicitly need to account for the geological, natural extent of human-modified and squeezed areas to fully understand their present-day developments. The geo-historical extent provides information on the long-term development of sedimentary structures and the functioning of coastal systems, and evaluates their effects on coastal protection, conservation and necessary adaptation. We use the Wadden Sea coastal zone as a case study to illustrate the necessity of the development of a geo-historical perspective and operationalize its integration into contemporary decision-making. The North European Wadden Sea is the largest continuous region of intertidal sand and mud flats in the world, and was recognized for its outstanding universal value by UNESCO in 2009. Nonetheless, it is an area that suffers intensely from coastal squeeze and has lost 70–80% of its coastal zone due to land reclamation and dike building. In short, our geo-historical perspective demands: (i) geo-historical risk assessment of coastal areas, (ii) geomorphological modelling that links the historical-induced risk to contemporary dynamics, (iii) assessment of ecological and societal values that are capable of estimating trade-offs between different coastal adaptation options in the contemporary system, (iv) evaluation of feasibility and legitimacy of potential coastal adaptation on the local scale of coastal inhabitants and (v) applying a transdisciplinary process that enables effective knowledge co-production between science and societal stakeholders.

## Impact statement

We emphasize the need for a geo-historical perspective in coastal protection planning and policy-making to tackle challenges presented by climate change, sea level rise, and biodiversity loss in densely populated coastal regions. Particularly affected are areas that have lost natural ecosystems due to land reclamation and dike construction. This perspective examines long-term changes in geomorphology and sedimentation from these human interventions.Our approach integrates advanced knowledge from various fields including geomorphology, numerical modelling, multi-criteria cost-benefit analysis, transdisciplinarity, and citizen empowerment. Using the Wadden Sea in Northern Europe as a case study, we argue for the necessity of expanding the discourse on coastal adaptation to incorporate a geo-historical lens. This involves reassessing the geographical boundaries for evaluating climate change effects, recognizing that previous paradigms often neglected areas beyond the dike line. Historically, the Wadden Sea encompassed larger regions that contributed to its ability to absorb storm surge energy, but this capacity has diminished due to human activities. To effectively integrate a geo-historical perspective, it is crucial to broaden the range of stakeholders in coastal protection and adaptation policy. This includes engaging citizens and decision-makers from inland areas. Such integration requires the development of inter- and transdisciplinary approaches and fostering a culture of constructive learning rather than political negotiation.We propose four archetypes of scenarios to facilitate the implementation of a geo-historical perspective. These scenarios should align with a transdisciplinary framework and include an action plan that acts as a boundary object for effective dialogue on coastal adaptation policies. Such tools are essential for addressing conflicts in perceptions and opinions in adaptation discussions.

## Introduction

The coastal challenges of the Anthropocene manifest in the search for new adaptation options, in particular for highly vulnerable (Armitage et al., 2025) and modified coastal zones (Vila-Concejo et al., 2024) considering related societal risks of climate change and sea level rise (Hermans et al., 2025). The necessity to redefine coastal adaptation measures and innovate long-term coastal protection policies, spatial planning and modern human settlement scenarios has become a salient issue in mandated authorities and among coastal communities worldwide (O’Riordan et al., 2008; Reise and MacLean, 2015; Philippenko and Le Cozannet, 2023; Hermans et al., 2025).

Typically, coastal zones have been of high economic interest, but were often in need of modifications like diking to protect settlements from storm surges and to fully utilize their economic potential. These processes have resulted in coastal squeeze and created coastal zone areas that are cut off from natural sedimentary dynamics (Delafontaine et al., 2000; Mai and Bartholomä, 2000; Vos and Knol, 2015; Karle et al., 2021). In times of climate change and sea level rise, coastal squeeze introduces particular challenges that are still often overlooked in contemporary planning and decision-making. This article offers a perspective on why historical, geomorphological knowledge should be integrated as an evidence base for coastal adaptation in squeezed coastal areas, and how such knowledge can be made relevant for long-term planning by both mandated decision-makers and local citizens.

The coastal zone of the Wadden Sea is hereby presented as a case study to illustrate the relevance of a geo-historical perspective and to operationalize its integration to contemporary decision-making. The Wadden Sea geomorphological system stretches from the Netherlands over Germany to Denmark. It has been designated for its Outstanding Universal Value as a UNESCO World Heritage site based on its function as a stopover in the Atlantic flyway, its function as a biodiversity hotspot and its unique dynamics in geological processes (Wehrmann, 2016). However, human influence has considerably changed and is continuously changing the geomorphological dynamics of the Wadden Sea through diking and other coastal protection measures (e.g. van der Spek, 1996; Reise, 2005; Oost et al., 2017; Karle et al., 2021). For example, in the German part of the Wadden Sea, the available space for geo-morphological dynamics has been reduced by 70–80% (Mai and Bartholomä, 2000; Karle et al., 2021) since humans started to build dikes. The Wadden Sea, in general, lost most of its transitional zone of salt marshes and reed belts.

Our perspective article is structured in the following sections. In Section “The historical–geomorphological development of the Wadden Sea and the impact of coastal squeeze”, we elaborate on the Wadden Sea historical–geomorphological development and how coastal squeeze impacted, and continues to impact, its geomorphological dynamics. In Sections “How can the historical–geomorphological perspective on the Wadden Sea alter future coastal management decisions and policy?” and “How to create societally relevant coastal adaptation scenarios?”, we explore how the knowledge about geo-historical dynamics can be made relevant for contemporary decision-making and long-term planning. To do so, in Section “How can the historical–geomorphological perspective on the Wadden Sea alter future coastal management decisions and policy?”, we propose four scenarios that help integrate geo-historical knowledge about the system understanding of the Wadden Sea. In Section “How to create societally relevant coastal adaptation scenarios?”, we elaborate on the contemporary governance system of the Wadden Sea and discuss how the knowledge generated by our scenarios can be made relevant not only for decision-makers but also for coastal inhabitants. In Section “Conclusions and take-home messages for the integration and operationalization of a geo-historical perspective”, we summarize the main take-home messages from our integration and operationalization exercise. Together, these take-home messages provide a roadmap for the integration of geo-historical knowledge into decision-making and long-term planning in the Wadden Sea, and can guide future scientific projects in coastal areas that also suffer from coastal squeeze.

## The historical–geomorphological development of the Wadden Sea and the impact of coastal squeeze

### Sea level rise and emergence of the Wadden Sea

The formation of the Wadden Sea began 7,000–8,000 years before present, when post-glacial sea level rise reached the region of the present-day Wadden Sea (Streif, 1987; Wehrmann, 2016). From a geomorphological viewpoint, the Wadden Sea area can be seen as a thin sediment wedge of Holocene age. This wedge marks the front of the post-glacial sea level rise, caused by the melting of ice sheets. Rising sea levels led to the reworking of older deposits, driven by both sea level rise and a lack of sediment input. The offshore edge, facing the North Sea, is characterized by strong currents that led, combined with storm surges and a mesotidal regime, to the initial formation of the barrier islands. Wind-blown sand, stabilized by vegetation, resulted in dune formation. Between the islands and the mainland, a broad transition zone with tidal flats, salt marshes and fens developed. Some North Frisian islands formed from erosion of older landforms and early salt marshes (Meier et al., 2013; Reise, 2013). To the southwest, similar processes formed the Zuiderzee, and peatland erosion connected the Almere Lake to the Wadden Sea, creating a large tidal basin (Berendsen, 2008).

### The land–sea transition zone: Components, processes and function

The Wadden Sea area before dike construction and land reclamation also included a wide land–sea transition zone. This area stretched along the coast and, due to extremely high tides and storm surges, also extended far inland. This semi-terrestrial environment was characterized by a variety of vegetation types, which functioned as ecosystem engineering species. The land–sea transition zone, therefore, typically developed as salt marshes, brackish lagoons and reed belts. Additionally, the saturated pore space in the adjacent terrestrial soils caused high groundwater tables, which favoured the formation of fens on the landward side of the transitional zone.

Due to the dense vegetation, two processes dominated the transitional zone: (i) fine-grained sediment accumulation by trapping and binding of suspended matter and (ii) formation of biogenic deposits by extensive production of plant biomass, primarily as organic-rich sediments or peat. Both processes resulted in net sedimentation and elevation of the topographic surface. In contrast, reworking or erosion was circumstantial. The absorption of hydrodynamic energy was another effect of the dense vegetation cover and successive heightening of the ground level. Therefore, the transitional zone also provided the ecosystem services of coastal protection, carbon storage and habitat diversity.

### Loss of the land–sea transition zone and its societal consequences

The human impact on the land–sea transition zone became significant with the onset of land reclamation and dike building. In the German part of the Wadden Sea coast, for example, a closed dike line has existed since the 13th century (Behre, 2008), decoupling nearly the entire transitional zone from sedimentary dynamics. Based on a comprehensive data set (LBEG, 2025), we reconstructed the paleo-landscape prior to the aforementioned land reclamation and dike building. The analysis revealed an area loss of 98% of the original land–sea transition zone.

The loss of the land–sea transition zone creates several societal problems, including coastal erosion, sediment deficits and ground subsidence. Originally, the natural processes of this zone were balanced with regional sea level changes and tidal forces, causing a long-term fluctuating coastline. However, by fixing the coastline with dikes, the hydrodynamic energy no longer dissipates gradually in the transition zone but hits the dike directly. As a result, fine sediments can no longer accumulate in sheltered areas and are washed out of the system. With rising sea levels, tidal flats and the dike foreland are becoming more frequently submerged. Although rising sea levels will create more space for sediment to accumulate, this space often will not be filled due to a lack of sediment (Colina Alonso et al., 2024; Miao et al., 2025). Offshore sand mining further intensifies this pressure. The buffer, which compensates for the lack of sediment, is shore face erosion in front of the barrier islands or deepening of the tidal channels (Pineda Leiva et al., 2025). However, with a long-term increase in hydrodynamic energy, the resource of appropriate sediment is being depleted, resulting in more frequent beach erosion.

In the land–sea transition zone of the Wadden Sea, a more detailed and synthesized understanding of the geographical manifestation of coastal erosion, sediment deficits and ground subsidence will be essential to calculate local levels of risk. Though much knowledge is available on land–sea transition, this knowledge is currently still scattered in a fragmented institutional landscape on the national and international level of the Wadden Sea area. Since climate change-induced sea level rise will increase the risks of flooding, storm surges and other coastal hazards, a synthesis of this knowledge will be important to understand which geographical areas are in particular vulnerable and due to which geomorphological characteristics.

The land–sea transition zones in squeezed coastal areas are thus much larger areas than what is typically identified as a coastal zone. Figure 1 indicates the coastal zone of the Wadden Sea area, defined as an area from −20 m water depth to +5 m elevation above sea level and demarcated by a red line.

**Figure 1.**
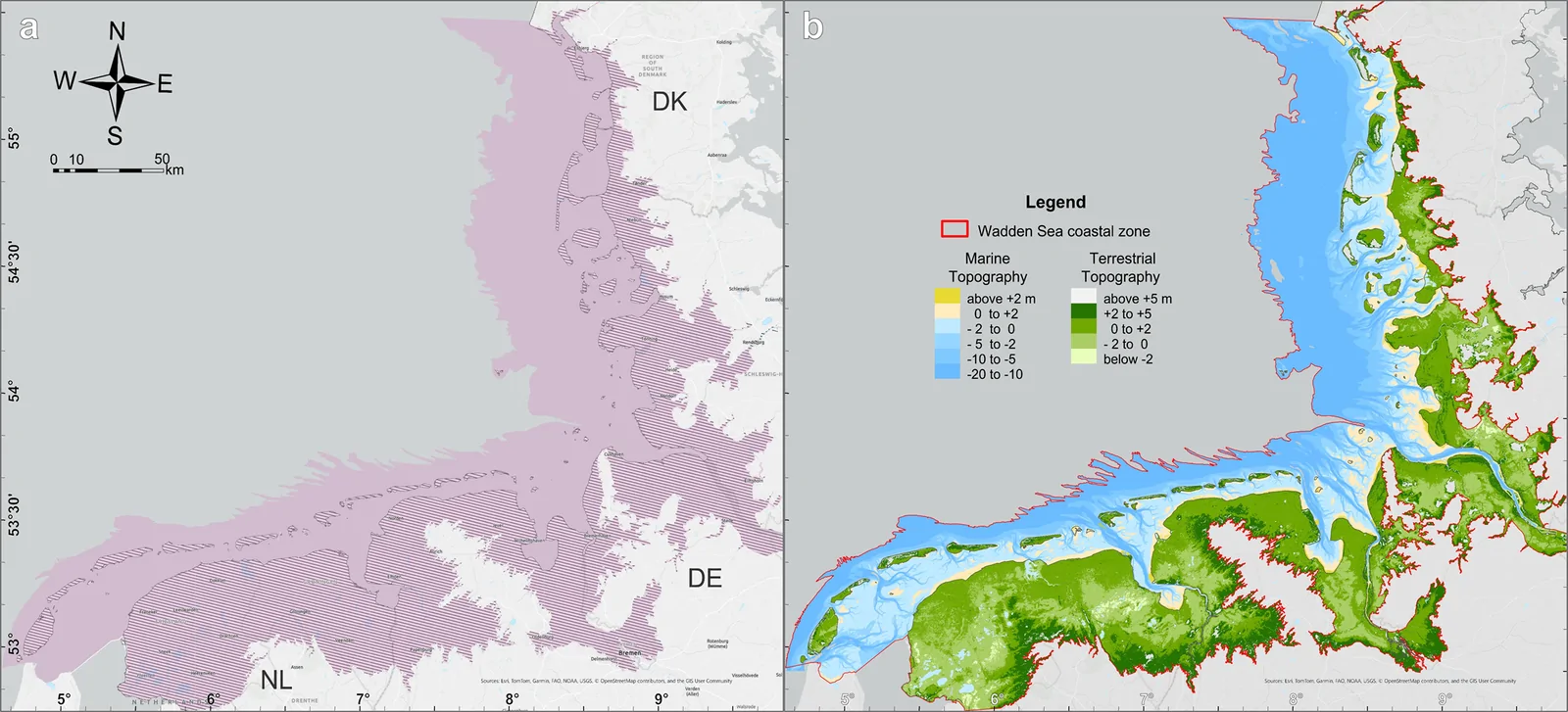
The Wadden Sea region. (a) The Wadden Sea coastal zone (highlighted in light red) in its spatial extension prior to dike building and land reclamation, extending across Denmark (DK), Germany (DE) and the Netherlands (NL). Present-day terrestrial areas are indicated by a hashed pattern. The Holocene sediment wedge is defined as the area between −20 m and +5 m relative to present mean sea level (= mid-tide level = NHN chart datum). (b) Topographic map of the Wadden Sea coastal zone. The light greenish colours indicate that today large areas of the mainland lie below high-tide level, as a long-term effect of decoupling from principal sedimentary processes since dike building. Basemaps were created using high-resolution digital elevation models, including Danmark’s Højdemodel (DHM; Danish Elevation Model), Actueel Hoogtebestand Nederland (AHN; Actual Elevation Levels for the Netherlands) and the Digitales Geländemodell (DGM1; Digital Terrain Model) for the German federal states of Niedersachsen, Schleswig-Holstein, Hamburg and Bremen. Marine topography data are from Milbradt and Pineda Leiva (2024), TrilaWatt: Topography (2015–2021), Federal Waterways Engineering and Research Institute. https://doi.org/10.48437/366eab-3640c8. Figure 1. Long description.

## How can the historical–geomorphological perspective on the Wadden Sea alter future coastal management decisions and policy?

To synthesize fragmented institutional knowledge and combine it with a geo-historical perspective, the development of scenarios is proposed as an effective strategy. Understanding coastal squeeze requires understanding the interaction among historical–geomorphological and current conditions. This could be achieved through sediment scenarios that allow for long-term sediment system behaviour to be interpreted into potential futures. The development of sediment scenarios, in conjunction with modelling studies (see e.g. Nienhuis and Lorenzo-Trueba, 2019), can provide a means of assessing how different coastal policies and changes in decision-makers’ frame of reference may impact the evolution of coastal systems (see e.g. Anarde et al., 2024). The goal of these types of studies is not to provide a definitive prediction of when, where and what sediment development will occur but to demonstrate how coastal systems respond to the implementation of changes in established policies and decisions, and to facilitate effective societal communication regarding coastal adaptation over the long term.

For our geo-historical perspective, we propose four different scenarios. Each scenario assesses an altered balance between historically induced geomorphological dynamics and (a change of) decisions about coastal protection measures. In addition, we will make use of different sea level rise scenarios, as current predictions still have a large uncertainty (Oppenheimer et al., 2019). These sediment scenarios under uncertain sea level rise are capable of integrating conjunctions with other components of the system, in particular the increase or decrease of biodiversity of the area, which is an important feature of the Outstanding Universal Value of the Wadden Sea.

### Scenarios for the future Wadden Sea

The *open boundary scenario* illustrates how nature could function with less constraint. This would force the land boundary and barrier shorelines to recede as sea levels rise, and transitional zones like salt marshes would increase, thus mitigating the effects of disconnection that had existed for many centuries due to land filling and the creation of dikes.

In the *nature-based solutions scenario*, there are calls for extensive restoration of the natural sedimentation and hydrodynamic processes, which help support resilient and adaptive environments and biodiversity (Temmerman et al., 2013; Wu et al., 2025). Though the strategy complies with the theory of resilience (Folke et al., 2004), there are challenges associated with the promotion and support of the safety and required standards (Moraes et al., 2022).

The *controlled dynamics scenario* melds classical engineering solutions, such as designed dike strengthening, with nature-based solutions like sediment improvement projects, such as artificial reefs. This strategy attempts to limit and control extremes, support ecosystem functioning and ensure multi-level safety standards are maintained (Schuerch et al., 2018).

In the *business-as-usual scenario*, it is assumed that there would be ongoing use of the traditional dike-line, dredge and hard engineering approach. This would limit sediment transport, enhance coastal squeeze and reduce ecological diversity, which would, in the end, cause degradation of cost-effectiveness and resilience despite the advantage of enhanced safety (Elschot et al., 2024).

Taken together, the above scenarios show how various boundary conditions, whether fixed, flexible or semi-restored, influence the future resilience of the Wadden Sea. Figure 2 summarizes the different effects of these scenarios on the geomorphological system of the Wadden Sea and its land-sea transition zone.

**Figure 2.**
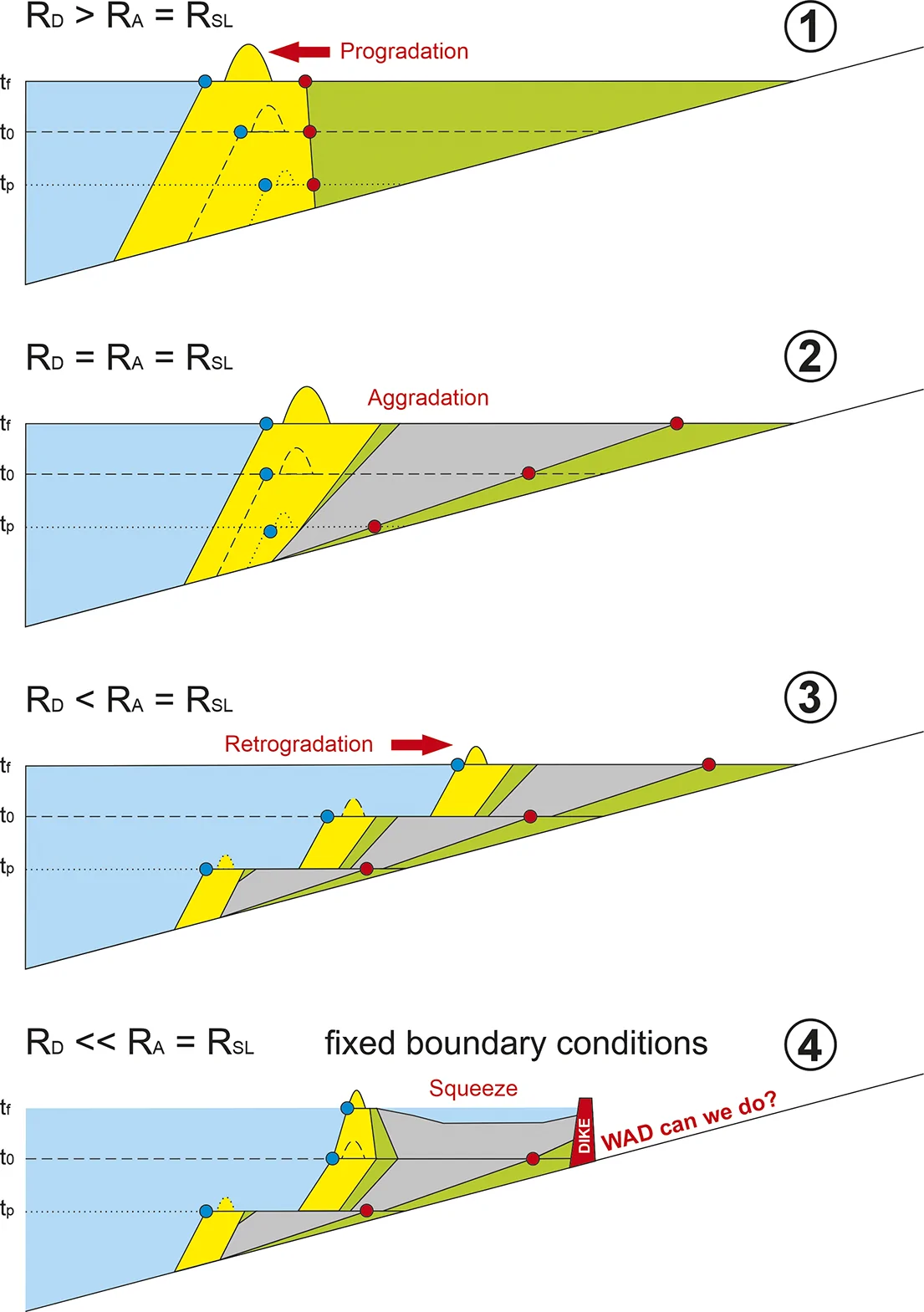
Simplified cross-section of the Wadden Sea sediment wedge under different spatio-temporal scenarios with respect to geo-historical variables. The dots mark the lateral shift of the outer Wadden Sea boundary (blue) and the coastline (red). The scenarios are based on the concept of sequence stratigraphy (Catuneanu, 2022), mainly controlled by the variables Rd. = deposition rate, Ra = accommodation rate, Rsl = sea level rise. The Ra reflects the potential space/volume (created by sea level rise) available for sedimentation. Green = transition zone, grey= tidal flats, yellow = dune island, *t*_p_ = past, *t*_0_ = present, *t*_f_ = future. As an example, scenario 3 would reflect the open boundary scenario under past and expected conditions, whereas scenario 4 reflects the business-as-usual scenario under historic and perspective fixed boundary conditions. Figure 2. Long description.

## How to create societally relevant coastal adaptation scenarios?

The integration of a geo-historical perspective into decision-making not only sets the demand for cross-institutional and cross-national knowledge synthesis and integration but it also urges a broadening of the type of discussion-partners currently involved in coastal policy adaptation and decision-making. The broadening of actor types would typically include public institutions that are not mandated for coastal policy, but operate within areas that are affected by potential adaptation solutions, for example, rural areas. Such areas have not gained much research attention so far in the realm of coastal adaptation, in particular when it comes to the search for nature-based adaptation solutions (Mortensen et al., 2024).

A transdisciplinary approach to coastal adaptation and policy-making is therefore required. Transdisciplinarity goes beyond traditional disciplinary boundaries by not only connecting different scientific fields but also actively involving stakeholders such as local communities and industry representatives (Pohl et al., 2021). Unlike multidisciplinary or interdisciplinary approaches, which mainly combine academic perspectives, transdisciplinarity creates a shared framework for understanding and problem-solving that bridges scientific knowledge with societal needs (Pohl and Hirsch Hadorn, 2007; Aeberhard and Rist, 2009; Pohl et al., 2021). By actively engaging diverse stakeholders in the co-design of solutions, transdisciplinarity not only ensures scientific soundness but also enhances social acceptance and political feasibility (Roux et al., 2010).

In that respect, coastal inhabitants are an important part of the geo-historical perspective, being strongly affected by climate change and sea level rise but also partly reinforcing those processes. Understanding and enhancing their adaptive capacity is necessary for successful adaptation. Individual adaptation refers to actions that individuals or households take to reduce the risks and negative impacts of environmental change (Carman and Zint, 2020). Such adaptation can be reactive or proactive and is influenced by psychological and social factors, including barriers such as self-efficacy and personal reactions, like hope or fear (van Valkengoed and Steg, 2019; Carman and Zint, 2020; Nalau and Verall, 2021). Moreover, it is necessary to examine adaptive behaviour in the local and individual context, as needs, conditions and available resources vary from place to place (Eriksen et al., 2011; Klöck, 2019). Adaptation measures are more likely to be implemented and effective when tailored to local conditions. Developing innovative approaches that facilitate and empower local citizens to understand and evaluate the effects and effectiveness of local adaptation measures in their personal context, therefore providing promising steps forward in increasing coastal adaptation in land–sea transition zones (Spencer et al., 2023).

Also, the historical understanding of human resource use, grown traditions and cultures of human settlements that have shaped the Wadden Sea landscape for centuries, in particular in the land–sea transition zone, but also the Hinterland (see e.g. Vollmer et al., 2001) gain renewed importance, as those provide important insights to local historical conditions, changes in the dominance of social values and the variety of human adaptation strategies that have been employed in the past (Thurstan, 2021). As such, the Wadden Sea cultural heritage can be seen as a pool of past experiences that provides important material for reflection upon future coastal adaptation options.

### Integrating a geo-historical perspective in the contemporary Wadden Sea governance system

The contemporary, cross-national Wadden Sea governance system is characterized by a long tradition of ecosystem-based management (EBM). The EBM approach is an environmental resource management strategy acknowledging the need for a holistic understanding of socio-ecological systems and sustainable resource use (McLeod et al., 2005). EBM promotes (scientific) knowledge as a key to planning and decision-making. The ecosystem-based management regime of the Wadden Sea emerged in the 1960s, pushed by prominent scientists and NGOs aiming to establish international cooperation in the Wadden Sea, capable of informing and influencing policies across national interests, based on the best knowledge available about the ecosystem(s) state and functioning (Olsen and Nickerson, 2003; Enemark, 2005). Defining characteristics of the Wadden Sea EBM include monitoring and assessment, adaptive management, collaborative planning and cross-jurisdictional coordination (EMI, 2019). An institutional backbone to the Wadden Sea EBM was established in 1987 in the form of the Common Wadden Sea Secretariat (CWSS), which facilitates and supports the trilateral exchange and synthesis of knowledge (Enemark, 2005). The CWSS regularly issues Quality Status Reports on the Wadden Sea to summarize, evaluate and communicate its overall development and ecological status (see Kloepper et al., 2017 for the latest QSR).

Understanding and connection of (scientific) research agendas and individual research projects’ results to policy- and decision-making is essential to keep the ecosystem-based management approach viable in the Wadden Sea area. With regard to sediment management, an important activity to support the contemporary governance system is to strengthen the already existing knowledge exchange and governance arrangements on the trilateral level. Although a lot of knowledge about the geomorphology of the Wadden Sea and related human activities, like dredging and resource extraction, is available (Kloepper et al., 2017; Schultze and Nehls, 2017), the debate on sediment dynamics has not yet reached an operational stage on the trilateral Wadden Sea level. So far, action is characterized by the synthesis of mainly national discussion and research projects. Further exploration of trilateral research gaps and needs, the definition of trilateral knowledge gaps between stakeholders and countries, the establishment of trilateral learning processes and knowledge exchange are needed.

To further develop, facilitate and strengthen the ecosystem-based trilateral dialogue and to integrate a geo-historical perspective, it is essential to understand the structure and functioning of the current sediment governance system. Key research questions need to address who the national decision-makers are, which sediment management strategies they apply, and whether – and to what extent – these sediment management strategies take their national and trilateral impacts on the state of the overarching Wadden Sea ecosystem and its Outstanding Universal Value into account. Are decision-makers actually aware of the potential of nature-based solutions (NBS) to increase the Outstanding Universal Value of the Wadden Sea? Based on which evidence is the effectiveness of nature-based solutions evaluated, and would national planning procedures allow the evaluation of the impact of NBS on a supranational level?

Governance system measuring methods like content and discourse analysis of policy documents, expert interviews and social network analysis are means to identify and analyse key decision-makers in the field and to analyse how knowledge streams (dis-)function. These types of analysis can be applied in the context of the trilateral Wadden Sea sediment governance system, to understand whether, how and on which scales actors in the system share knowledge, perceive similar knowledge gaps, have different or contradictory knowledge and see potential for realizing the actual harmonization of sediment knowledge in the form of adapted measurements, parameters or adapted policies and decision-making procedures.

Case study evidence of the development of recent coastal adaptation policies in German parts of the Wadden Sea indicates that key decision-makers have acknowledged the merit of, but on the organizational level had problems in effectuating cross-institutional and cross-disciplinary collaboration (Walsh, 2019). It became clear that cleavages between nature conservation and coastal protection are persistent and bound not only at the personal, legal and institutional but also at the technical levels (Walsh, 2019; Kreis and Prominski, 2025). The organization of any type of transdisciplinary dialogue will therefore benefit from the use of a boundary object that clarifies such cleavages. A boundary object does not intend to change existing social values, but uses a common reference point to clarify different perceptions and opinions about an issue (Star and Griesemer, 1989; Becker, 2017; Beck et al., 2021). The neutral position of a scientist can benefit this process and could, for example, be seized through the development of a regional action plan that clarifies a common need for coastal adaptation and identifies persistent cleavages that hinder a transdisciplinary debate.

### Developing an action plan as a boundary object

For the integration of a geo-historical perspective, an action plan could be used to understand how different actor groups in the research area perceive the historical boundary of the Wadden Sea, whether and to what extent this boundary already is integrated into their policies and decision-making, and why or why not it would be necessary or possible for them to integrate it? In this context, the application of a transdisciplinary evaluation scheme would be an important means to keep track of the action plan development process. A transdisciplinary evaluation scheme can be used to identify individual participants’ struggles in the integration of a geo-historical perspective and can identify and manage conflicts before they negatively affect the process. To do so, the transdisciplinary evaluation scheme would need to analyse both effective and ineffective knowledge integration on the cognitive level of individual participants and innovatively integrate emotional and social–interactional dimensions to capture the full complexity of transdisciplinary engagement (Giebels et al., 2020; Pohl et al., 2021).

Figure 3 features a roadmap to the integration of a geo-historical perspective to future coastal adaptation and policy-making in squeezed systems and summarizes its main building blocks.

**Figure 3.**
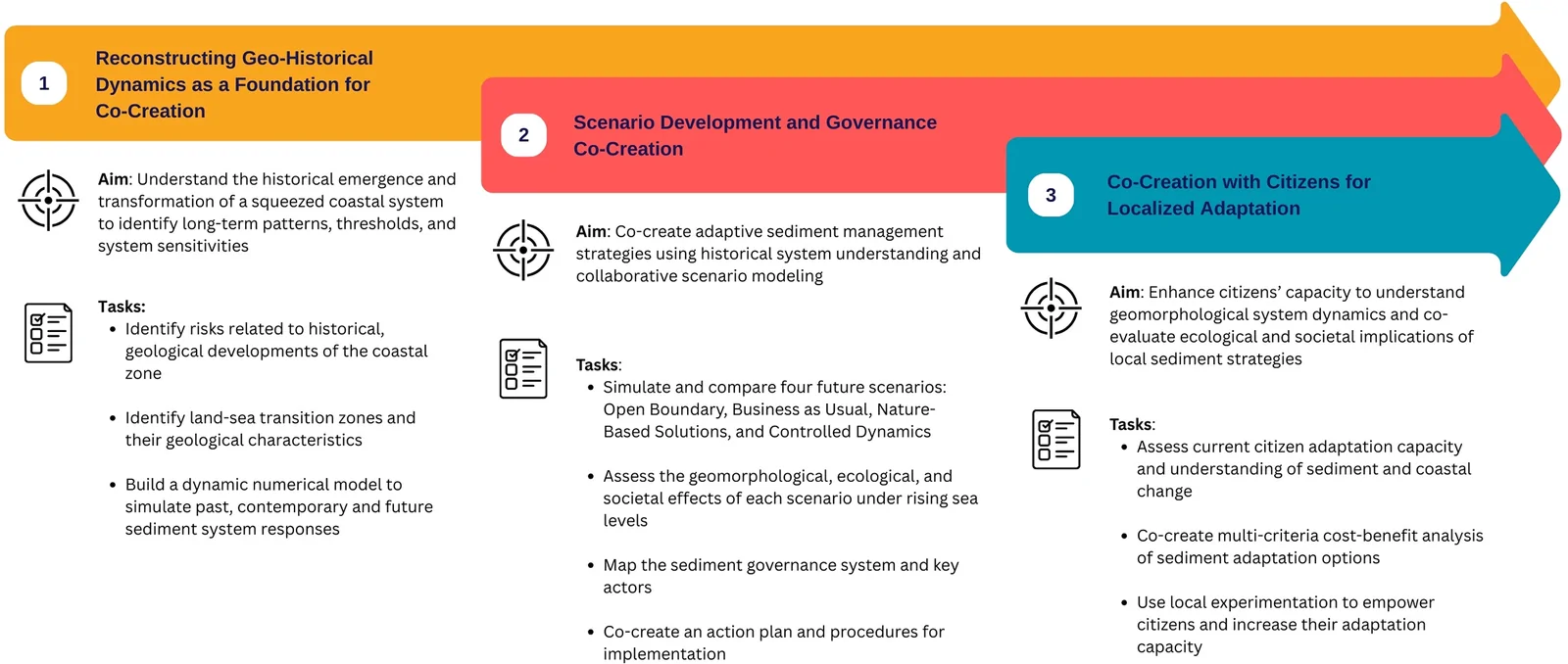
Roadmap for integration of a geo-historical perspective to future coastal adaptation and policy-making in squeezed systems. Figure 3. Long description.

## Conclusions and take-home messages for the integration and operationalization of a geo-historical perspective

Our perspective expands the contemporary debate about coastal adaptation policies and measures, with the need to design adaptation and mitigation options for geographical areas that are currently not part of the coastline but represent land–sea transition zones. To integrate and operationalize a geo-historical perspective for these areas, which are typically characterized by a fixed coastline and a low-lying coastal plain, important steps are:

- Define focus areas along squeezed coastal zones in which the pressure for adaptation from the geo-historical perspective and related risks like, for example, unstable grounds are the highest. Such historical, geomorphological knowledge is easily overlooked in contemporary decision-making organizations, as they tend to focus on mobile sediment that can be used as a building resource. However, immobile, fixed geomorphological structures also affect the overall dynamics of complex coastal systems and, therefore, need to become part of their overall understanding and adaptive management.
- Model morphological dynamics of the coastal system and relate those to the risks derived from the geo-historical understanding of the coastal zone. The modelling process can help identify the different cause-and-effect relationships in a system. If designed in a transdisciplinary way, the model results can also facilitate stakeholder integration and co-production, clarifying and eventually harmonizing diverging or fragmented views on the system.
- Evaluate the impact of changing morphological dynamics on the overall ecological and societal values represented in the area. Impact evaluation can be used as a means to integrate geo-historical knowledge with available knowledge about the current state of the coastal zone.
- Develop future scenarios for future coastal change that integrate and hence are able to consider varying priorities for ecosystems, human activities and resilience of the coastal zone. Scenarios are an efficient way to integrate stakeholders and to reflect upon the relevance of a geo-historical perspective on contemporary decision-making. They can also be used to understand whether and to what extent different management options will improve either ecology, social liveability or other human activities of a coastal area.
- Assess the feasibility and legitimacy of potential coastal change on the local scale of coastal inhabitants. Since a geo-historical perspective on a squeezed coastal area by definition includes human settlement areas that are not necessarily linked to direct ocean dynamics anymore, it is important that awareness, attitude and capacity towards coastal adaptation on the scale of individuals, households and communities is facilitated and empowered beyond coastal areas.
- Organize, design, facilitate and evaluate the transdisciplinary knowledge generation process between science and societal stakeholders. Boundary objects, like the development of an action plan, could assist this process. The change of polices about coasts and adjacent regions will require changes in the mindsets of decision-makers, citizens and other resource users alike, and typically come along with conflicts. While conflict resolution can be seen as a main task for the political arena, science nonetheless can assist in the development of boundary objects, like an action plan, that synthesizes knowledge from different sources and identifies knowledge cleavages or gaps.

## Data Availability

Data availability is not applicable to this article as no new data were created or analysed in this study.

## Long descriptions

### Figure 1. Long description

Panel a shows the historical spatial extension of the Wadden Sea coastal zone highlighted in light red. The area extends from D K in the North, through D E in the East and South, to N L in the Southwest. Present-day terrestrial areas within this zone are marked with a horizontal hashed pattern. A compass rose in the top left indicates North, and a scale bar shows 0 to 50 kilometers.

Panel b is a topographic map of the same region with a detailed legend. The Wadden Sea coastal zone is outlined by a red boundary. Marine topography is shown in shades of blue and yellow: yellow for above plus 2 m, and light to dark blue for depths ranging from 0 to minus 20 m. Terrestrial topography is shown in shades of green: dark green for above plus 5 m, medium green for plus 2 to plus 5 m, light green for 0 to plus 2 m, and pale yellow-green for areas below minus 2 m. Large sections of the mainland, particularly in the German and Dutch sectors, are shown in light green and pale yellow-green, indicating they currently lie below high-tide level.

Navigate back to Figure 1..

### Figure 2. Long description

A four-panel cross-section diagram illustrating sediment wedge dynamics. Each panel features a vertical axis with three time steps: t sub p (past), t sub 0 (present), and t sub f (future). Blue dots mark the outer boundary and red dots mark the coastline.

* Scenario 1: R sub D is greater than R sub A which equals R sub S L. A yellow dune island moves seaward (left) over time, labeled Progradation with a left-pointing red arrow. The green transition zone expands seaward.

* Scenario 2: R sub D equals R sub A which equals R sub S L. The yellow dune island and grey tidal flats stack vertically, labeled Aggradation. The boundaries remain relatively stable laterally.

* Scenario 3: R sub D is less than R sub A which equals R sub S L. The yellow dune island and grey tidal flats shift landward (right) as sea level rises, labeled Retrogradation with a right-pointing red arrow.

* Scenario 4: R sub D is much less than R sub A which equals R sub S L under fixed boundary conditions. A red vertical block labeled DIKE prevents landward migration. The grey tidal flat area is compressed between the rising sea and the dike, labeled Squeeze. Red text asks W A D can we do?

Navigate back to Figure 2..

### Figure 3. Long description

The flowchart consists of three staggered, right-pointing arrows labeled 1 through 3.

Stage 1 is an orange arrow titled Reconstructing Geo-Historical Dynamics as a Foundation for Co-Creation. Its aim is to understand the historical emergence and transformation of a squeezed coastal system to identify long-term patterns, thresholds, and system sensitivities. Tasks include identifying risks related to historical geological developments, identifying land-sea transition zones, and building a dynamic numerical model for sediment system responses.

Stage 2 is a pink arrow titled Scenario Development and Governance Co-Creation. Its aim is to co-create adaptive sediment management strategies using historical system understanding and collaborative scenario modeling. Tasks include simulating four future scenarios (Open Boundary, Business as Usual, Nature-Based Solutions, and Controlled Dynamics), assessing effects under rising sea levels, mapping the sediment governance system, and co-creating an action plan.

Stage 3 is a teal arrow titled Co-Creation with Citizens for Localized Adaptation. Its aim is to enhance citizens’ capacity to understand geomorphological system dynamics and co-evaluate ecological and societal implications of local sediment strategies. Tasks include assessing current citizen adaptation capacity, co-creating multi-criteria cost-benefit analysis of adaptation options, and using local experimentation to empower citizens.

Navigate back to Figure 3..
