# Supplementary material for: The key to our future lies in the past: How to integrate historical–geomorphological knowledge for future coastal decision-making?
Source: Camb Prism Coast Futur. 2026 Apr 23;4:e24. doi: 10.1017/cft.2026.10029 (PMC13748881; doi:10.1017/cft.2026.10029)
Supplement: Peer review history

# Reviewer report: Review: The key to our future lies in the past: How to integrate historical–geomorphological knowledge for future coastal decision-making? — R0/PR2

**DOI:** 10.1017/cft.2026.10029.pr2

General Comments

The manuscript addresses a relevant and timely issue. However, in its current form, the structure and focus make it difficult to follow. The paper contains valuable information, but the main argument is somewhat diluted by excessive technical detail and the lack of a clear narrative thread.

As far as I can understand, this is a Perspective article. Therefore, I suggest reorganizing the manuscript to follow a clearer and more concise structure: starting with an introduction that clearly states the objectives, followed by a single section on the Wadden Sea case study, and ending with a concluding section summarizing the key take-home messages.

Specific Suggestions

- Clarify the overall aim of the paper at the end of the Introduction. The Introduction correctly sets up the main issue, but the scope and objectives are not clearly presented. You may conclude the Introduction with a sentence such as: “This Perspective argues that …. We use the Wadden Sea as a case study to illustrate how …...”

- You could merge Sections 2 and 3 into a single section titled “The Wadden Sea as a case study.”

- Reduce technical detail in the middle sections and focus instead on conceptual insights relevant to your main proposal. The sections on sediment dynamics and modelled scenarios appear too detailed for a Perspective and would fit better in a research article.

- Strengthen the transitions between sections to improve the narrative flow.

- Add a short Conclusions section summarizing the key messages and implications for future coastal policy and research. The article currently lacks a proper closing section; in a Perspective, readers usually expect final take-home messages and an outlook for future directions.

# Editor report: Recommendation: The key to our future lies in the past: How to integrate historical–geomorphological knowledge for future coastal decision-making? — R0/PR3

**DOI:** 10.1017/cft.2026.10029.pr3

**Affiliations:** Flinders University, Australia

Please carefully consider the reviewers suggestions and critique

# Editor report: Decision: The key to our future lies in the past: How to integrate historical–geomorphological knowledge for future coastal decision-making? — R0/PR4

**DOI:** 10.1017/cft.2026.10029.pr4

**Affiliations:** Geography, Cambridge University, United Kingdom of Great Britain and Northern Ireland

No accompanying comment.

# Reviewer report: Review: The key to our future lies in the past: How to integrate historical–geomorphological knowledge for future coastal decision-making? — R1/PR6

**DOI:** 10.1017/cft.2026.10029.pr6

This paper makes a valuable contribution to advancing the understanding of the relevance of past geomorphological dynamics for coastal adaptation at the Waden Sea but with application more broadly.

Section 2: This section is could be improved through aditional references - e.g. Reise (2013) on the natural history of the Wadden Sea is a key reference for historical perspective, perhaps also Meier et al (2013) Der Küstenatlas: Das schleswig-holsteinische Wattenmeer in Vergangenheit und Gegenwart. Extensive reference is made in this section to the historical Wadden Sea without sufficient recognition of the dynamic nature of this landscape. When is referred to? At different times in the past, swimming peatlands were quite extensive at parts of the coast, for example. Does the paper refer to a time before dike-building or before human settlement. Greater precision here would be helpful, particularly as conservationists often refer to a historical, primordial Wadden Sea in an unreflective manner (see also the critique in Reise 2013 above).

Recent paper on “Asymmetric morphodynamics of the Wadden Sea” is very relevant re. ongoing legacy of past geomorphological changes and human interventions.

Section 3: Here it would be helpful to refer to existing studies that have examined future scenarios under climate change at the Wadden Sea (e.g Reise 2015 Kurswechsel Küste, Kreis, D. & Prominski, M. (2025) GAIA among others. Very relevant here is that the climate adaptation strategy for the Wadden Sea in S-H (Wattenmeer 2100) deliberately excluded any discussion of the dikeline and the lands behind the dikes (see Walsh (2019) Ocean and Coastal Management). In a strategy with a long-time horizon, thinking beyond the dikes as a fixed boundary was not possible.

p. 18, Lin3 343-360 - The governance implications here could be elaborated on further and made more concrete. For example, a key current question is how nature-based solutions in coastal management can be aligned with the Wadden Sea conservation objectives, specifically the OUV of the World Heritage site.

The conclusions are very focused and practical. They could be elaborated further with perhaps some general conclusions of the relevance of the study beyond the Wadden Sea. It could also be interesting to consider what a geohistorical perspective means in interdisciplinary terms - e.g. inclusion of historical archival material from a humanities perspective or is natural science analyses sufficient. There are cognate developments in the interdisciplinary field of marine historical ecology (e.g. Thurstan 2022), and authors such as Linde Egberts have previously called for a nuanced historical perspective on the Wadden Sea landscape to inform future practice.

Minor comments:

p. 2 line 45 - “informs on” is not correct English. Perhaps ‘informs how we understand’, or similar

p3 line 97 - should be ‘ integration into contemporary’

p3 line 107 ‘zone of by salt marshes’ - delete ‘by’ ?

p 4 line 130 - barrier islands established - needs rephrasing - became established perhaps?

There may be further typographical / language issues further on in the article that I have not picked up.

# Reviewer report: Review: The key to our future lies in the past: How to integrate historical–geomorphological knowledge for future coastal decision-making? — R1/PR7

**DOI:** 10.1017/cft.2026.10029.pr7

**Authors:** Karsten Reise

**Affiliations:** Alfred-Wegener-Institut Helmholtz-Zentrum für Polar- und Meeresforschung, Germany

Suggestions of the reviewer have been suffiently followed. I agree, that the squeezed coastal zone of the Wadden Sea requires a transdisciplinary approach with sound knowledge of the past to arrive at sustainable adatations to accelerating sea level rise.

Some minors may be considered:

L 40 should be: ... on how historical AND geomorphological ...

Fig 1 contains inconsistencies: (1) too many red lines in the NE and SW and one missing at Bremerhaven, (2) clour code for <0 m in the sea and on land should be the same, (3) lakes and reservoirs should be colloured light blue and not grey.

Fig 2 Explain symbols of the abcissa and explain what ‘accommodation rate’ refers.

# Editor report: Recommendation: The key to our future lies in the past: How to integrate historical–geomorphological knowledge for future coastal decision-making? — R1/PR8

**DOI:** 10.1017/cft.2026.10029.pr8

**Affiliations:** Geography, Cambridge University, United Kingdom of Great Britain and Northern Ireland

Good alignment here over minor revision

# Editor report: Decision: The key to our future lies in the past: How to integrate historical–geomorphological knowledge for future coastal decision-making? — R1/PR9

**DOI:** 10.1017/cft.2026.10029.pr9

**Affiliations:** Geography, Cambridge University, United Kingdom of Great Britain and Northern Ireland

No accompanying comment.

# Editor report: Recommendation: The key to our future lies in the past: How to integrate historical–geomorphological knowledge for future coastal decision-making? — R2/PR11

**DOI:** 10.1017/cft.2026.10029.pr11

**Affiliations:** Geography, Cambridge University, United Kingdom of Great Britain and Northern Ireland

Revision 2 now acceptable. Accept.

# Editor report: Decision: The key to our future lies in the past: How to integrate historical–geomorphological knowledge for future coastal decision-making? — R2/PR12

**DOI:** 10.1017/cft.2026.10029.pr12

**Affiliations:** Geography, Cambridge University, United Kingdom of Great Britain and Northern Ireland

No accompanying comment.
